# A Redox-Switchable Supramolecular Carbohydrate Chip for Capture and Real-Time Glycosylation Analysis of Living Suspension Cells

**DOI:** 10.3390/biom16101404

**Published:** 2026-09-26

**Authors:** Yuchao Lu, Qian Li, Juan Liu, Xian Chen, Yuxin Pei, Zhongfu Wang, Teodor Aastrup, Changping Hu, Yanbin He, Zhichao Pei

**Affiliations:** 1 Shanxi Provincial Department-Municipal Key Laboratory Cultivation Base for Quality Enhancement and Utilization of Shangdang Chinese Medicinal Materials, School of Pharmacy, Changzhi Medical College, Changzhi 046000, China; 2 Chemical Biology, College of Chemistry and Pharmacy, Northwest A&F University, Yangling 712100, China; 3 College of Life Science, Northwest University, Xi’an 710069, China; 4 Attana, SE-11419 Stockholm, Sweden

**Keywords:** host–guest systems, supramolecular carbohydrate chips, quartz crystal microbalance, carbohydrate–protein interactions, glycosylation analysis

## Abstract

Real-time analysis of aberrant glycosylation on living suspension cancer cells is critical for early cancer detection and therapeutic development, yet the lack of non-destructive immobilization methods for these cells limits the advancement of cell-based biosensors. Herein, a supramolecular carbohydrate chip (SMCC) is presented that enables efficient capture of living cancer cells via multivalent glycan–protein interactions. Based on redox-switchable ferrocene–pillar[6]arene host–guest chemistry, this platform supports real-time binding kinetic analysis of lectin–glycan interactions on cell surfaces under physiological conditions. The chip can be regenerated electrochemically, allowing multiple assay cycles while preserving cell viability. This approach provides a versatile tool for dynamic glycosylation monitoring and cancer biomarker research, with potential applications in drug discovery and clinical diagnostics.

## 1. Introduction

Aberrant glycosylation on cancer cell surfaces is a hallmark of tumorigenesis, metastasis, and immune evasion [1,2] and serves as a critical target for cancer diagnosis and therapeutic intervention [3,4,5]. As one of the most common post-translational modifications, glycosylation affects over 70% of human proteins and plays essential roles in protein folding, stability, trafficking, and cell–cell communication [6]. In cancer, altered glycosylation—manifested as increased N-glycan branching, elevated sialylation, and expression of truncated O-glycans such as Tn and sialyl-Tn antigens—is closely associated with malignant transformation and metastatic progression [7]. These changes influence tumor cell adhesion, migration, immune evasion, and resistance to apoptosis. Consequently, real-time and in situ analysis of glycosylation on living cancer cell surfaces is critical for understanding tumor biology and for developing new diagnostic and therapeutic strategies [8].

Over the past few decades, several technologies, such as surface plasmon resonance (SPR) and quartz crystal microbalance (QCM), have been developed to study the expression of glycosylation on cancer cells’ surfaces [9,10,11]. SPR monitors refractive index changes near a sensor surface with high sensitivity, while QCM detects mass changes through resonance frequency shifts. However, these methods often rely on covalent immobilization of bio-probes (e.g., lectins or antibodies), which leads to irreversible binding and static interfaces, thereby limiting their capability for dynamic monitoring and sensor regeneration [12,13,14,15]. Covalent attachment also complicates probe removal without surface damage and restricts the study of reversible or sequential interactions. Such limitations hinder the ability to dynamically monitor the spatiotemporal heterogeneity of glycosylation on suspension tumor cells, particularly in complex biological environments [16,17,18]. Moreover, suspension cells do not naturally adhere to surfaces, and conventional immobilization methods are often too harsh for living cells. Therefore, a gentle and reversible cell capture strategy is required to enable repeated measurements on the same cell population.

Supramolecular chemistry, leveraging dynamic host–guest interactions, offers a promising strategy to address these challenges by enabling redox-switchable molecular recognition, surpassing static physical or covalent immobilization approaches [19,20]. Through non-covalent interactions such as host–guest recognition, hydrogen bonding, and metal coordination, supramolecular systems can be designed to respond to external stimuli, enabling reversible assembly and disassembly. The ferrocene–pillar[6]arene system is particularly attractive for biosensing applications. The binding affinity between ferrocene and pillar[6]arene can be modulated electrochemically: oxidation of ferrocene to ferrocenium markedly reduces the binding constant, allowing guest dissociation under mild conditions [21]. This redox responsiveness enables surface regeneration without harsh chemicals or extreme pH, which is crucial for maintaining cell viability. Unlike covalent bonds, ferrocene–pillar[6]arene host–guest complexes exhibit redox-triggered reversible binding, enabling real-time interfacial adaptability and sensor regeneration under mild conditions [22]. The programmable multivalency of pillar[6]arenes allows spatial organization of glycan ligands into high-density clusters, mimicking natural glycan–protein interactions and enhancing binding affinity by up to 100-fold through avidity effects, as demonstrated in other multivalent macrocyclic systems [23,24]. This multivalent presentation is particularly effective for capturing cells, as cell-surface receptors often engage in multiple simultaneous interactions with clustered ligands. Importantly, these systems eliminate complex chemical modifications and simplify chip regeneration compared to covalent strategies [25].

To further enhance surface functionality, polydopamine (PDA) was employed for its superior adhesion and antifouling properties [26]. PDA can be deposited onto diverse substrates through simple dip-coating in alkaline dopamine solution. The versatility of PDA coatings has made them a popular choice for constructing biosensing interfaces, particularly for cell-based assays where biocompatibility and surface functionality are critical. Its catechol and amine groups enable secondary reactions, including Michael addition and Schiff base formation, for immobilizing guest molecules or biomolecules. Compared to static covalent immobilization, dynamic host–guest chemistry enables reversible sensing, yet existing systems lack gentle regeneration and multivalent live-cell capture. Our work integrates a redox-switchable ferrocene–pillar[6]arene with PDA to achieve reversible, multivalent cell capture and real-time glycosylation analysis—a combination not yet demonstrated in QCM cell sensors. This design offers several distinct advantages: PDA provides a stable yet functional adhesion layer; the pillar[6]arene scaffold enables multivalent glycan presentation that mimics natural glycoconjugate clustering; and electrochemical regeneration operates under physiological conditions, preserving cell viability and supporting multiple assay cycles. Recently, Chen et al. reported a photo-switchable glycochip for light-controlled cell capture [27]; our redox-switchable platform offers complementary advantages, including electrochemical regeneration without UV irradiation and real-time lectin–glycan kinetic profiling. Herein, we present a multivalent supramolecular carbohydrate chip via host–guest chemistry for label-free and real-time monitoring of abnormal glycosylation on tumor cells. This work sits at the intersection of supramolecular chemistry, interface materials science, and real-time bioanalysis. Polydopamine (PDA)-functionalized substrates were employed to immobilize ferrocene guest molecules (see synthesis and characterization in Supplementary Figures S1–S3) through Michael addition, followed by the assembly of N-acetylglucosamine-functionalized pillar[6]arene (GlcNAc-P[6]) (see synthesis and characterization in Supplementary Figures S4–S8) via host–guest recognition [28]. The multivalent interactions between chip surface carbohydrates and cell surface proteins were exploited to capture suspension tumor cells onto the chip surface, producing a suspension tumor cell chip (see Scheme 1) [29,30,31].

## 2. Materials and Methods

### 2.1. Reagents and Instruments

Dopamine hydrochloride, concanavalin A (Con A), D-galactose, tris(hydroxymethyl)aminomethane (Tris) were purchased from Sigma-Aldrich (St. Louis, MO, USA). Wheat germ agglutinin (WGA), Ulex europaeus agglutinin I (UEA-I), and soybean agglutinin (SBA) were purchased from Vector Labs (Burlingame, CA, USA). Hexanediamine, N-acetylglucosamine (GlcNAc), D-mannose, and L-fucose were obtained from Sarn Chemical Technology (Shanghai, China). Hydroquinone and brominated peracetylated N-acetylglucosamine were obtained from Aike (Chengdu, China) and bromopropyne from Heowns (Tianjin, China). Ferrocene carboxylic acid was obtained from Energy Chemical (Shanghai, China) and the fluorescent stain Hoechst 33258 from Solaibio (Beijing, China) Human acute T lymphocytic leukemia cells (Jurkat) and human chronic myelogenous leukemia cells (K562) were generous gifts from Prof. Bin Gao (Institute of Microbiology, Chinese Academy of Sciences, Beijing, China).

NMR spectra were recorded on a Bruker Avance III spectrometer (Bruker Corporation, Billerica, MA, USA) with a nominal ^1^H frequency of 500 MHz. ^1^H NMR spectra were acquired at 500 MHz, and ^13^C NMR spectra at 125 MHz. Chemical shifts are reported relative to residual solvent signals (D_2_O: ^1^H δ 4.79 ppm; CDCl_3_: ^1^H δ 7.26 ppm, ^13^C δ 77.16 ppm). Gold sensor chips with 10 MHz AT-cut quartz crystals were obtained from Attana AB. Biosensor experiments were performed using an Attana Cell A200 QCM instrument (Attana AB, Stockholm, Sweden) at 20 °C with pH 7.4 PBS as running buffer.

### 2.2. Synthesis of Ferrocene–Hexane Diamine and N-GlcNAc-Functionalized Pillar[6]arene

Full synthetic procedures and characterization data (NMR and MS spectra) are provided in the Supporting Information (Figures S1–S8). The ferrocene–hexane diamine guest molecule and the N-GlcNAc-functionalized pillar[6]arene host molecule were synthesized according to the routes shown in the SI. Briefly, the peracetylated 2-azido-GlcNAc derivative (**H1**) was prepared by nucleophilic substitution of brominated peracetylated N-acetylglucosamine with NaN_3_ in DMF at 80 °C for 12 h. The alkynylated pillar[6]arene **H2** was synthesized by BF_3_·OEt_2_-catalyzed condensation of 1,4-bis(prop-2-yn-1-yloxy)benzene with paraformaldehyde in CHCl_3_ at room temperature. Copper-catalyzed azide–alkyne cycloaddition (CuAAC) between **H1** and **H2** afforded the peracetylated intermediate **H3**. The final deacetylated product (GlcNAc-P[6]) was obtained by NaOMe/MeOH deprotection of **H3**, and its structure was indirectly confirmed by the subsequent host–guest assembly and cell-capture experiments. The synthesis and characterization of **H1**–**H3** are detailed in the Supplementary Information.

### 2.3. Preparation of PDA-Based Supramolecular Carbohydrate Chip

The chip surface was first cleaned three times with 10 mL of 1% NaClO for 2–3 min each time. Then, the chip was cleaned twice with ultrapure water. Next, 3 mL of concentrated sulfuric acid was slowly added along the wall of the vessel and gently shaken to cover the chip. After that, 1 mL of 30% H_2_O_2_ solution was slowly added dropwise to create a piranha solution, and the chip was soaked in this solution for 20 min. Following the soaking, the chip was rinsed with ultrapure water, washed with anhydrous ethanol 2–3 times, and dried with nitrogen (N_2_).

Secondly, the PDA chips were fabricated by preparing a 2 mg/mL dopamine hydrochloride solution using 10 mM pH 8.5 Tris-HCl buffer and incubating 50 μL dropwise on the surface of each chip for 1.5 h at room temperature to allow its self-agglomeration. Then, 10 mM, 50 μL of the ferrocene–hexane diamine guest molecule was attached to the chip surface by Michael addition occurring via amino groups to the PDA chip, incubated for 2 h at room temperature, washed with ultrapure water, and dried with N_2_.

Thirdly, the ferrocene–hexane diamine-modified PDA chip was incubated with 15% H_2_O_2_ for 4 min to obtain the oxidized ferrocene group, rinsed with ultrapure water, and then dried with N_2_. Then, 50 μL of 10 mM N-GlcNAc-functionalized pillar[6]arene host molecules were immobilized onto the chip surface by host–guest interaction, incubated for 2 h at room temperature, and then washed with ultrapure water to produce supramolecular carbohydrate chips.

### 2.4. Characterizations

The thickness and surface roughness of supramolecular carbohydrate chips were measured with an atomic force microscope (AFM) (Bruker, Billerica, MA, USA). The chemical composition of the chip’s surface was determined by scanning electron microscopy X-ray energy spectrometry (FE-SEM) (Hitachi, Tokyo, Japan).

### 2.5. Preparation of Living Suspension Cell Chips

Jurkat and K562 cells were cultured in RPMI 1640 medium containing 10% FBS and 1% penicillin-streptomycin. Cells in the logarithmic growth phase were taken and diluted to 1.4 × 10^6^ cells/mL using pH 7.4 PBS buffer to prepare cell suspensions. The modified supramolecular carbohydrate chips were placed in a 24-well plate with 0.5 mL (7 × 10^5^ cells) of Jurkat or K562 cell suspension and incubated in a cell incubator (37 °C with 5% CO_2_) for 1.5 h. The cells were immobilized by the glyco-cluster effect of the N-GlcNAc groups on the carbohydrate chip surface with the suspension cell surface proteins. Then, the chip was washed 2–3 times with PBS to remove the unfixed cells, and the cells on the chip surface were stained with Hoechst 33258 Living Cell Staining Solution. The growth state of the cells, as well as their coverage, was observed with fluorescence microscopy (Olympus BX53, Olympus Corporation, Tokyo, Japan). After each QCM detection, the cells on the chip surface were stained again and evaluated with fluorescence microscopy.

### 2.6. QCM Detection and Data Collection

The cell chip was prepared and inserted into the Attana Cell biosensor. A pH 7.4 PBS solution was used as the running buffer, and the chip was stabilized at a flow rate of 20 μL/min with a frequency change of less than 5 Hz in 600 s. To block the remaining uncovered glycosyl groups and PDA on the chip surface, a 100 μg/mL bovine serum albumin (BSA) solution was injected 3–5 times until the frequency signal stabilized, indicating that non-specific binding sites were saturated. Following this, a 50 μg/mL lectin solution was injected, and the binding time of lectin to cell surface glycans was 105 s, with a dissociation time of 295 s.

For kinetic experiments, different concentrations of lectin (100, 50, 25, and 12.5 µg/mL) prepared in PBS were introduced onto the chip surface for detection. Subsequently, a high concentration (300 mM) of the corresponding monosaccharide solution was injected to elute the lectin for 15 s, thus regenerating the cell chip. Other lectins were then injected to detect the level of different glycol units on the surface of Jurkat and other cells. Each lectin was tested at least three times to reduce errors. The resonance frequency changes in the suspension cell chip were measured and recorded in real-time by Attester software (Attester v3.1, Attana AB, Stockholm, Sweden). The data from competition experiments were fitted with GraphPad Prism 8, and the experimental kinetic data were fitted with Clamp XP version 3.50.

### 2.7. Regeneration of Chips

The cell chip was placed in a 24-well plate, and 0.5 mL of a 20 mM mercaptoethanol solution prepared in PBS was added. It was then incubated at room temperature for 30 min to allow the reduction of the oxidized ferrocene group. After this, the chip was disassembled with pillar[6]arene to remove the cells and achieve regeneration of the chip. Subsequently, the mercaptoethanol solution was removed, and the chip surface was washed with PBS 2–3 times. The cells on the chip surface were stained with Hoechst 33258 and observed by fluorescence microscopy to ensure that the cells on the chip surface had been completely removed.

## 3. Results and Discussion

### 3.1. Preparation of the Supramolecular Carbohydrate Chip-Based Living Suspension Cell Biosensor

The PDA chip and the PDA-based supramolecular carbohydrate chip were characterized using AFM and SEM-EDS. The AFM analysis revealed that the thickness of the PDA-modified gold chip significantly increased compared to the bare gold chip, with a maximum thickness of 86.5 nm and the average roughness (Ra, defined as the arithmetic average of the absolute deviations of the surface profile from the mean line) increasing from 1.04 nm to 15.8 nm (Figure 1a–c). This roughness increase is characteristic of PDA film formation via dopamine self-polymerization under alkaline conditions. The resulting three-dimensional structure offers a large surface area and abundant reactive sites for subsequent ferrocene immobilization. After assembly of the GlcNAc-P[6] host, the surface became more undulating, with the maximum height reaching 194.8 ± 21.2 nm and Ra increasing to 39.5 ± 4.8 nm (Table S1). The further increase in roughness after host assembly suggests that the pillar[6]arene macrocycles form a well-defined layer rather than simply aggregating on the surface. The multiple binding sites of pillar[6]arene likely promote uniform distribution of the host molecules. These results confirm the successful layer-by-layer construction of the supramolecular architecture.

SEM-EDS spectra of the PDA chip and the PDA-based carbohydrate chip were recorded (Figure 1d,e). The EZAF quantification showed that the Fe signal appeared only on the carbohydrate chip (0.38 at%), confirming successful immobilization of the ferrocene guest. The N content increased from 22.16 at% on the PDA chip to 28.50 at% on the carbohydrate chip, consistent with the introduction of the N-containing ferrocene guest and GlcNAc-P[6] host. The C content did not increase significantly (27.53 vs. 26.47 at%), which is reasonable because both PDA and the carbohydrate layer contain carbon. Given that EDS is semi-quantitative for light elements, these elemental changes are used as supporting evidence together with AFM and control experiments.

The functional performance of the supramolecular glycochip was rigorously evaluated through quantitative cell capture experiments. As shown in Figure S9, the chip efficiently captured Jurkat cells via multivalent glycan–protein interactions between the surface GlcNAc clusters and cell surface lectin-like receptors. Hoechst 33258 staining of all nuclei gave an initial captured cell density of 1182 ± 265 cells/field. To demonstrate reusability, the chip was subjected to two complete regeneration and recapture cycles. After the first regeneration using mercaptoethanol reduction, the capture efficiency remained at 94.7% (1119 ± 237 cells/field), and after the second regeneration, 74.4% of the initial capture capacity was retained (880 ± 61 cells/field). The gradual decrease in capture efficiency over successive cycles is likely attributable to several factors, including incomplete reassembly of the ferrocene–pillar[6]arene complexes after each regeneration cycle, minor irreversible oxidation of ferrocene during the electrochemical treatment, and accumulation of residual cellular material or adsorbed protein on the chip surface. These factors are common in reversible supramolecular systems and can be mitigated through further optimization of the regeneration conditions.

Cell viability throughout the operational cycle was quantitatively assessed via live/dead assays (Figure S10). Live/dead staining showed that 707 ± 158 of the captured cells were live immediately after capture. After QCM detection, 614 ± 24 live cells/field remained, corresponding to 86.8% viability retention relative to the post-capture live cell number (614/707). After regeneration and re-capture, 645 ± 33 live cells/field were observed. The difference between the Hoechst count and the live-cell count is because Hoechst stains all nuclei, including dead or compromised cells, whereas the live/dead assay counts only membrane-intact live cells. These results confirm that the electrochemical treatment and chip regeneration do not significantly harm the cells. Maintaining cell viability is essential for ensuring that the measured glycosylation profiles reflect the native state of the cells rather than stress-induced artifacts.

Control experiments confirmed the specificity of the capture mechanism. Negligible cell adhesion was observed on chips modified only with PDA or PDA–ferrocene (without GlcNAc-P[6]) (Figure S11), ruling out non-specific adsorption and confirming that efficient capture is specifically mediated by the multivalent glycan interactions enabled by the host–guest assembly. The specificity is further supported by the release of captured cells upon ferrocenium reduction, which disrupts the host–guest complex and removes the multivalent glycan display from the surface.

### 3.2. Evaluation of Suspension Tumor Cell Surface Glycosylation

To demonstrate the analytical capability of the platform, we evaluated the glycosylation profiles of Jurkat and K562 cells using four different lectins: WGA (recognizes GlcNAc and sialic acid), Con A (recognizes mannose/glucose), SBA (recognizes N-acetylgalactosamine, GalNAc), and UEA-I (recognizes L-fucose). Each lectin targets a distinct glycan epitope, enabling a comparative assessment of glycan abundance across the two cell lines. The binding and dissociation processes were monitored in real-time by QCM, providing both qualitative and quantitative information about the interactions.

Figure 2a shows a representative sensorgram for WGA binding to K562 cells, demonstrating that the chip could be regenerated after each lectin injection and maintained reproducible response signals. The nearly identical response amplitudes from three consecutive WGA injections indicate that the chip surface can be reliably regenerated after each binding cycle, which is essential for quantitative comparisons across different lectins and cell lines. The maximum frequency response values of WGA for both cell types were apparently higher than those of the other three lectins, at 98.5 Hz and 56.7 Hz for Jurkat and K562 cells, respectively (Figure 2b). This suggests that GlcNAc-containing glycans are more abundant on the surfaces of these cells than mannose/glucose-, GalNAc-, or fucose-containing structures. It should be noted that the frequency responses reflect not only the abundance of specific glycan epitopes but also their accessibility, spatial presentation and multivalency on the cell surface. The lectin binding data should therefore be interpreted as relative indicators of glycan availability rather than direct measures of glycan abundance. It should also be noted that WGA recognizes both GlcNAc and sialic acid residues. The WGA binding response therefore reflects the combined contribution of these two glycan epitopes. While this limits the specificity of WGA as a probe for GlcNAc alone, it does not affect the overall conclusion that the glycosylation profiles of Jurkat and K562 cells are apparently different.

Notably, the frequency response values of Con A showed only a slight difference between the two cell types, indicating a similar content of mannose/glucose-containing glycan structures on both cell lines. In contrast, the responses from SBA and UEA-I were relatively low for both cell types, suggesting lower expression levels of GalNAc and fucose-containing glycans. Jurkat and K562 cells are both hematopoietic cell lines, but they exhibit distinct glycosylation patterns. Previous studies have reported that K562 cells express higher levels of GlcNAc-terminated glycans, whereas Jurkat cells show relatively higher expression of mannose/glucose-containing glycans. These differences are consistent with our observations and support the validity of our platform for detecting cell-surface glycosylation heterogeneity. The results demonstrate that the frequency response values of different lectins varied between the two cell types, indicating apparent differences in surface glycosylation between K562 and Jurkat cells.

### 3.3. Competition and Inhibition Experiments

To further validate the specificity of lectin–glycan interactions on the chip, competitive inhibition experiments were performed using D-mannose as a competitor for Con A binding. Before each experiment, 50 µg/mL of Con A was mixed with varying concentrations of D-mannose (0–5 mM) and incubated for 30 min. The corresponding mannose solution was first injected as a blank control, followed by injection of the Con A/mannose mixture. The maximum frequency shift during binding was recorded as the amount of Con A bound under inhibition.

As shown in Figure 3a, the binding of Con A decreased with increasing concentration of D-mannose, demonstrating the competitive inhibitory effect of the free monosaccharide. The binding at 0 mM mannose was set to 100%, and the data were fitted to a four-parameter logistic inhibition model (Equation (S1)). The IC_50_ value of 2.2 mM should be regarded as an apparent half-inhibitory concentration that reflects the overall binding behavior under the multivalent conditions of the assay. It provides a useful comparative parameter but should not be interpreted as an intrinsic dissociation constant. This result confirms that the lectin binding observed on the chip is indeed specific and that the QCM platform can be used for quantitative inhibition studies.

### 3.4. Kinetic Evaluation

The QCM-based cell biosensor enabled real-time monitoring of biomolecular interactions on living cell surfaces, providing insights into binding kinetics and interaction mechanisms. The interactions between WGA, Con A, and the glycan epitopes on Jurkat and K562 cells were analyzed using a concentration series of each lectin (12.5, 25, 50, and 100 µg/mL) at 20 °C. The sensorgrams for each concentration were globally fitted to a 1:1 Langmuir binding model that includes a mass transport term, accounting for both lectin diffusion to the surface and the binding reaction itself.

Figure 3b shows representative sensorgrams for WGA binding to Jurkat cells. All response curves were globally fitted to a 1:1 binding model with mass transport using ClampXP version 3.50 to derive the kinetic rate constants, including the association rate constant (*k_on_*), dissociation rate constant (*k_off_*), and equilibrium dissociation constant (*K_D_* = *k_off_/k_on_*) (Table 1). The close agreement between the experimental data and the fitted curves confirms that the 1:1 model adequately describes the binding behavior. The *K_D_* values reported here should be regarded as apparent or effective dissociation constants that reflect the overall avidity of the multivalent lectin–glycan interactions on the cell surface, rather than intrinsic molecular affinities.

The kinetic parameters reveal distinct glycosylation characteristics between the cell lines. The apparent *K_D_* value for WGA binding to K562 cells (0.81 nM) was lower than that for Jurkat cells (2.80 nM), suggesting a possible trend of stronger WGA avidity toward K562 cells. However, because these values are apparent parameters derived from a single global fit and the cell-surface glycome is multivalent and heterogeneous, this difference should not be over-interpreted. The Con A responses for Jurkat and K562 cells were similar and within experimental error, and no statistically significant difference can be claimed from the present dataset. These apparent kinetic differences nevertheless suggest that the glycosylation landscapes of Jurkat and K562 cells differ in ways that merit further investigation with independent replicates and more selective lectins. Notably, the differences in *K_D_* between the two cell lines cannot be fully explained by glycan abundance alone—as estimated from the maximum frequency shifts—indicating that glycan presentation and accessibility also play critical roles in determining binding affinity. These kinetic differences provide quantitative evidence that the glycosylation landscapes of Jurkat and K562 cells differ not only in the abundance of specific glycans but also in the presentation and accessibility of these structures, which governs lectin binding affinity and complex stability. Such quantitative kinetic information is essential for understanding the molecular basis of glycan-mediated recognition events and for evaluating the functional consequences of glycosylation changes in disease states. This kinetic profiling underscores the capability of our QCM platform to resolve both quantitative and qualitative differences in cell surface glycosylation.

## 4. Conclusions

In summary, we have developed a reversible and reusable supramolecular carbohydrate chip (SMCC) that enables non-destructive capture of living suspension cancer cells for real-time QCM analysis of cell surface glycosylation. The integration of PDA-based surface engineering with redox-switchable host–guest chemistry provides a robust and dynamic platform for assessing carbohydrate–protein interactions under physiological conditions. The platform offers several key advantages: (1) efficient multivalent capture of living suspension cells without covalent fixation; (2) real-time, label-free kinetic analysis of lectin–glycan interactions with quantitative binding parameters; (3) electrochemical regeneration enabling multiple assay cycles (74.4% capture retention after two cycles); and (4) preservation of cell viability throughout the operational cycle (>86% viability retention). This regenerability substantially enhances experimental throughput and cost-effectiveness.

We acknowledge that further optimization of the host–guest interface is needed to improve long-term stability, as capture efficiency declines with repeated regeneration. Moreover, while the chip performs well with cultured cell lines, its application to clinical samples—such as circulating tumor cells—will require validation in more complex biological matrices. Nevertheless, this work establishes a versatile analytical tool with significant potential for investigating glycosylation-related biomarkers and screening glycan-targeting drugs. Future work will employ more selective lectins, such as sialic acid-specific lectins, to further dissect the contributions of individual glycan types. While the present study focused on two leukemia cell lines to establish proof of concept, future work will include healthy cell lines and primary cells to further validate the differential glycosylation analysis. With continued development, this supramolecular carbohydrate chip could become a valuable tool for glycosylation analysis in both basic research and clinical settings.

## Figures and Tables

**Scheme 1:**
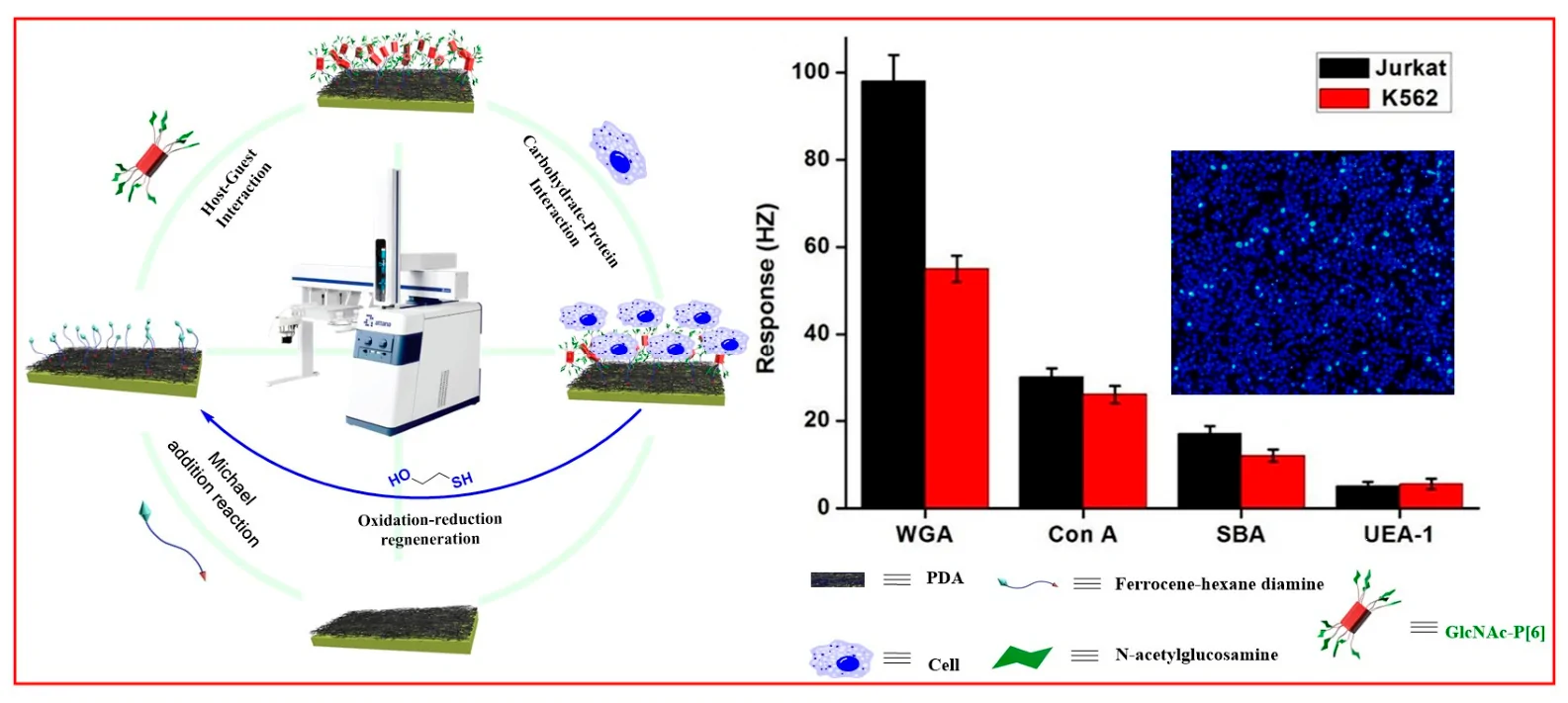
Illustration of the supramolecular carbohydrate chip prepared by host–guest interaction, which can successfully capture living suspension tumor cells for fabricating QCM living suspension cell biosensors.

**Figure 1:**
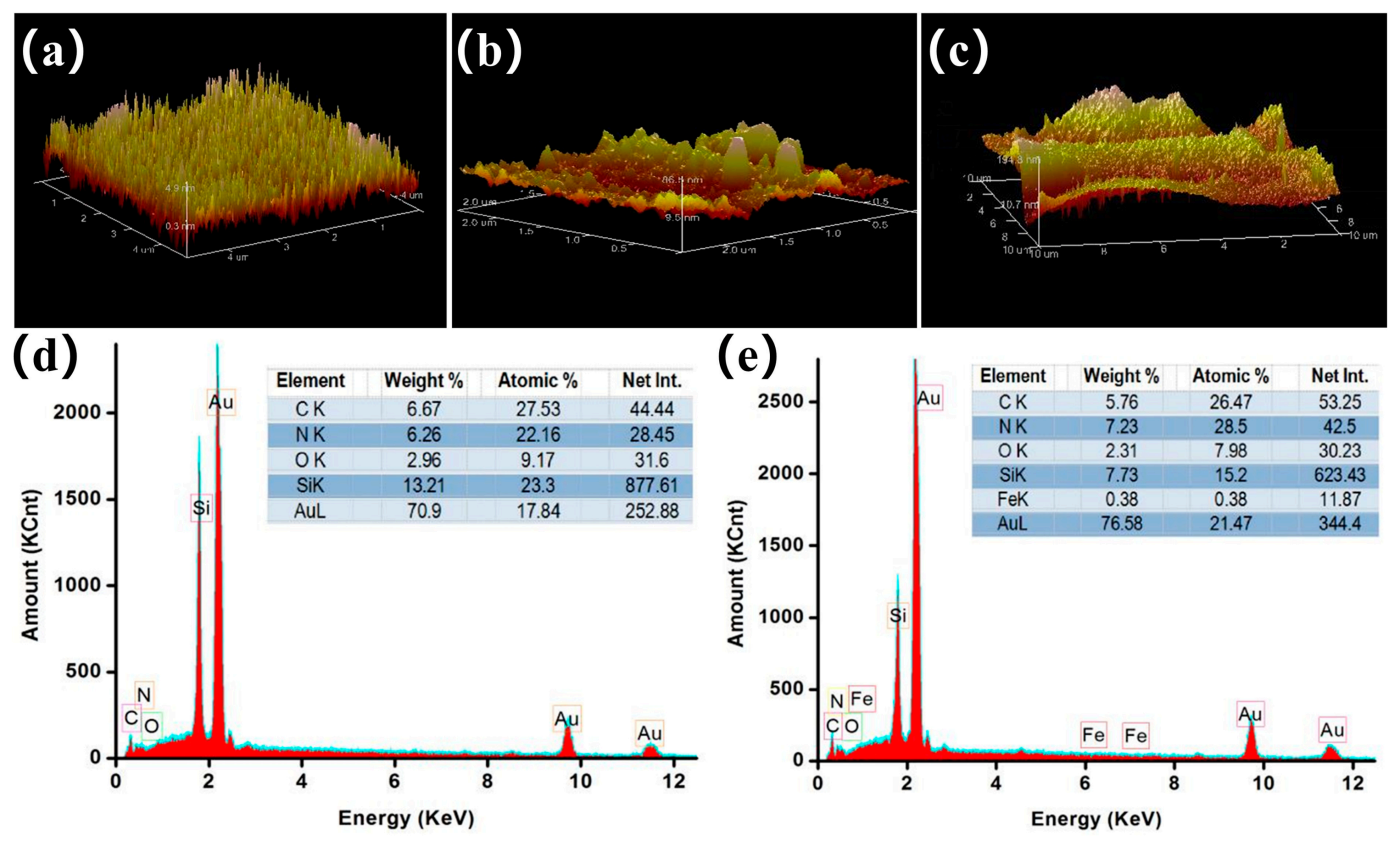
The 3D surface morphology of AFM (**a**) gold chip; (**b**) PDA chip; (**c**) carbohydrate chip; (**d**) SEM-EDS spectra of PDA modified chip surface; (**e**) carbohydrate chip.

**Figure 2:**
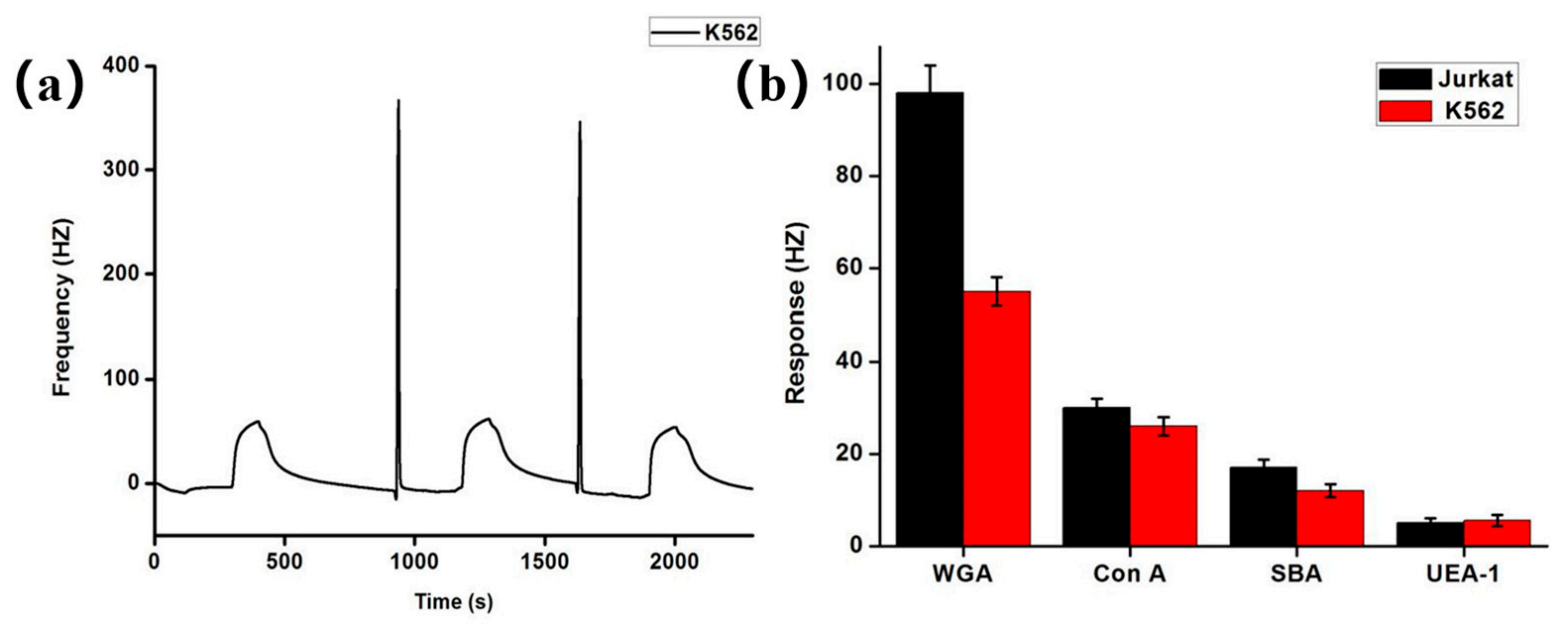
(**a**) The frequency shift was recorded during the binding and regeneration of WGA to K562 cells on QCM sensor surfaces; (**b**) lectins elicited responses from Jurkat cells and K562 cells. WGA, Con A, SBA, and UEA-I at 50 μg/mL were introduced to the suspension-cell sensor surfaces, respectively. The maximum frequency shift in each lectin was recorded and normalized to cell coverage. The data represent 3 independent experiments from 3 chips and are presented as mean ± s.d.

**Figure 3:**
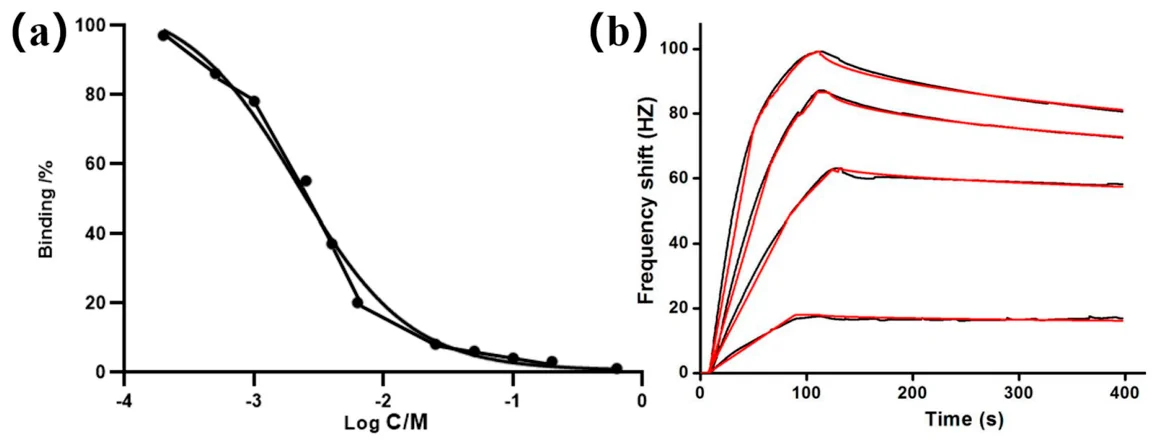
(**a**) Competitive inhibition of the interaction of Con A with Jurkat cells using D-mannose as the competitor. The x-axis represents the logarithm of the mannose concentration (log C/M); (**b**) Kinetic evaluation of the interaction of WGA with Jurkat cells. WGA at 12.5, 25, 50, and 100 μg/mL were tested. The responses were recorded (black curves) and were globally fitted to a theoretical 1:1 binding model with mass transport parameters (red curves).

**Table 1:** Apparent kinetic parameters for WGA and Con A binding to Jurkat and K562 cells.

Lectin	Cell Line	*k_on_* (M^−1^ s^−1^)	*k_off_* (s^−1^)	*K_D_* (nM)
WGA	Jurkat	1.12 × 10^6^	3.14 × 10^−3^	2.80
	K562	1.26 × 10^6^	1.02 × 10^−3^	0.81
Con A	Jurkat	1.06 × 10^4^	2.05 × 10^−5^	1.94
	K562	0.74 × 10^4^	0.93 × 10^−5^	1.26

## Data Availability

The original contributions presented in this study are included in the article/Supplementary Material. Further inquiries can be directed to the corresponding authors.

## Supplementary Materials

The following supporting information can be downloaded at https://www.mdpi.com/article/10.3390/biom16101404/s1: Figure S1: Figure S1: ^1^H-NMR spectrum of Compound G1. Figure S2: ^1^H-NMR spectrum of Compound G2. Figure S3: ^13^C-NMR spectrum of Compound G2. Figure S4: ^1^H-NMR spectrum of Compound H1. Figure S5: ^1^H-NMR spectrum of Compound H2. Figure S6: ^1^H-NMR spectrum of Compound H3. Figure S7: ^13^C-NMR spectrum of Compound H3. Figure S8: HRMS spectrum of Compound H3. Figure S9: Fluorescence micrographs of Jurkat cells captured on the supramolecular glycochip before and after regeneration. Figure S10: Live/dead fluorescence staining of Jurkat cells on the supramolecular glycochip across key processing stages. Figure S11: Fluorescence images of cells on (a) PDA-modified chip and (b) PDA-ferrocene-modified chip (without GlcNAc-P[6]). Table S1: AFM measurement of chip 3D surface morphology: roughness and thickness comparison (n = 3). References cited in the Supplementary Materials [8,28,32,33,34].
