# Supplementary material for: A Redox-Switchable Supramolecular Carbohydrate Chip for Capture and Real-Time Glycosylation Analysis of Living Suspension Cells
Source: Biomolecules. 2026 Sep 26;16(10):1404. doi: 10.3390/biom16101404 (PMC13702469; doi:10.3390/biom16101404)
Supplement: Supplementary file 1 [file biomolecules-16-01404-s001.zip › biomolecules-4555395-supplementary.pdf]

Supplementary Information

A Redox-Switchable Supramolecular Carbohydrate Chip for Capture and Real-Time Glycosylation Analysis of Living Suspension Cells

Yuchao Lu [1,*,†], Qian Li [2,†], Juan Liu [1,†], Xian Chen [2], Yuxin Pei [2], Zhongfu Wang [3], Teodor Aastrup [4], Changping Hu [1], Yanbin He [1] and Zhichao Pei [2,*]

[1] Shanxi Provincial Department-Municipal Key Laboratory Cultivation Base for Quality Enhancement and Utilization of Shangdang Chinese Medicinal Materials, School of Pharmacy, Changzhi Medical College, Changzhi 046000, China

[2] Chemical Biology, College of Chemistry and Pharmacy, Northwest A&F University, Yangling 712100, China

[3] College of Life Science, Northwest University, Xi'an 710069, China

[4] Attana, SE-11419 Stockholm, Sweden

* Correspondence: luyuchao@czmc.edu.cn (Y.L.); peizc@nwafu.edu.cn (Z.P.)

† These authors contributed equally to this work.

Table of Contents

1. Synthesis and characterizations of compounds... S2
2. Cell culture.. S5
3. Preparation and characterizations of Chip... S5
4. Fitting equation.. S10
5. Figure S1-S11.. S11
References S15

1. ***Synthesis and characterizations of compound***

Synthesis of **Ferrocene derivative**

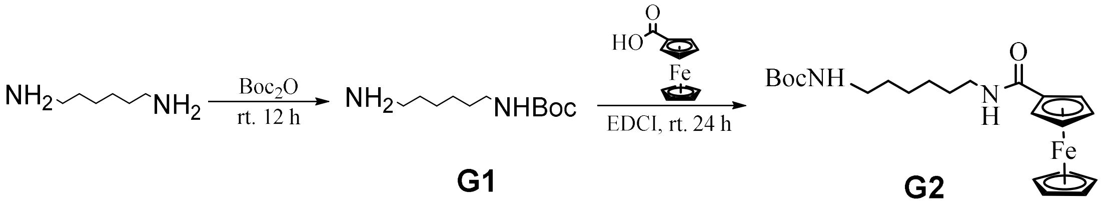

G1 **G2**

(1) Compound **G1**

Add 1.8 g hexanediamine and 15 mL of THF (dry) to a 100 mL round bottom flask. At 0°C, 0.88 g Boc_2O was dissolved in 10 mL THF (dry) and slowly added dropwise to the reaction solution over 30 min. The reaction was carried out at room temperature for 12 h. Upon completion of the reaction as monitored by TLC, the solvent was removed under reduced pressure, 100 mL ethyl acetate was added and washed three times with saturated sodium chloride solution (3×50 mL), and the combined organic phases were dried with anhydrous magnesium sulfate. After filtration, the solvent was removed under reduced pressure, and the white solid compound **G1** was obtained by direct column separation. The yield was 89%.

(2) Compound **G2**

Add 0.37 g ferrocenecarboxylic acid, 0.32 g compound **G1**, 0.3 g EDCI and 10 mL dichloromethane to a 100 mL flask and react under N_2 protection for 24 h. After complete reaction by TLC, 50 mL dichloromethane was added and washed three times with saturated sodium chloride solution (3×50 mL), and the organic phase was dried with anhydrous sodium sulfate. After filtration, the solvent was removed under reduced pressure and the light yellow solid compound **G2** was obtained by direct column separation. The yield was 88%. ^1H NMR (500 MHz, $CDCl_3$) δ/ppm= 6.00 (s, 1H), 4.70 (s, 2H), 4.59 (s, 1H), 4.34-4.29 (m, 2H), 4.21 (d, J = 23.6 Hz, 5H), 3.35 (dd, J = 13.2, 6.7 Hz, 2H), 3.13 (d, J = 6.2 Hz, 2H), 1.62-1.55 (m, 2H), 1.51-1.47 (m, 2H), 1.44 (s, 9H), 1.36 (dd, J = 22.6, 9.8, 6.8 Hz, 4H).

Synthesis of **N-GlcNAc-functionalized pillar[6]arene**[1-3]

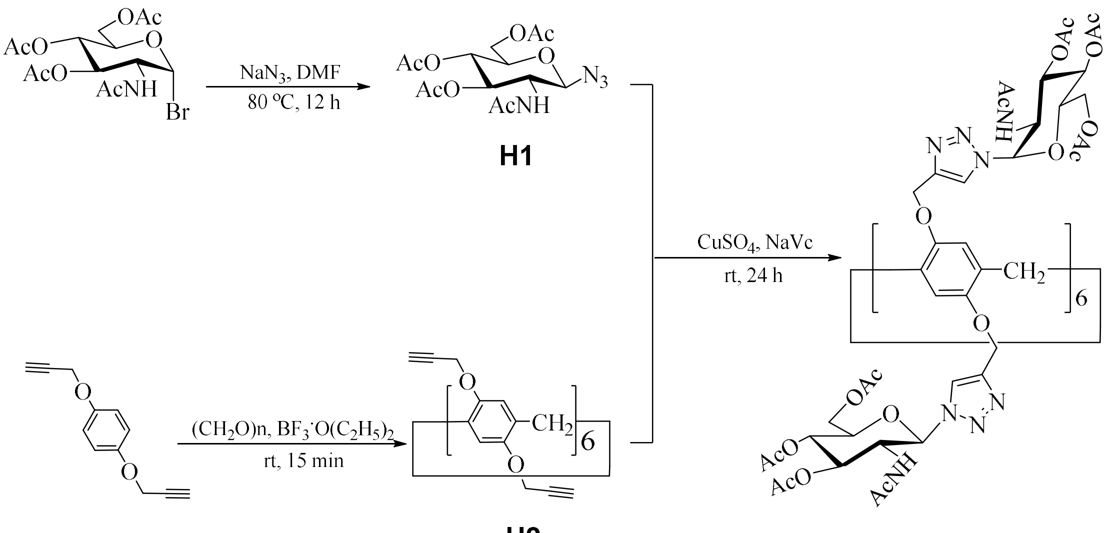

(1) Compound **H1**

Brominated peracetylated N-acetylglucosamine (2.01 g, 0.005 mol) and sodium azide (0.636 g, 0.01 mol) were dissolved in DMF (3 mL), and the mixture was stirred at 80 °C for 12 h under N_2. After completion, the reaction was cooled, poured into ice water (30 mL), and extracted with ether (5 mL × 3). The organic layer was dried over anhydrous Na_2SO_4, filtered, and evaporated to give compound **H1** as a white solid in 91% yield.

(2) Compound **H2**

1,4-Bis(prop-2-yn-1-yloxy)benzene (2.72 g, 20 mmol) was dissolved in chloroform (60 mL). Paraformaldehyde (1.398 g, 40 mmol) was added, and the mixture was stirred at room temperature for 15 min. $BF_3 \cdot OEt_2$ (4 mL) was then injected, and the reaction was continued at room temperature for 15–20 min. After completion, the mixture was poured into ice water (150–200 mL), quenched, and filtered. The collected solid was extracted with saturated $NaHCO_3$, deionized water, and saturated NaCl. The organic layer was dried over anhydrous Na_2SO_4, filtered, and purified by column chromatography to give compound **H2** in 17% yield. ^1H NMR (500 MHz, $CDCl_3$) δ/ppm=6.84 (s, 12H), 4.54 (d, J = 2.4 Hz, 24H), 3.86 (s, 12H), 2.44 (t, J = 2.3 Hz, 12H).

(3) Compound **H3**

Compound **H1** (500 mg, 1.33 mmol) and compound **H2** (66 mg) were dissolved in CH_2Cl_2 (10 mL). Copper sulfate pentahydrate (30 mg, 0.12 mmol), sodium ascorbate

(80 mg, 0.57 mmol), and deionized water (10 mL) were added sequentially at room temperature under N_2. The mixture was stirred for 24 h. The organic layer was dried over anhydrous Na_2SO_4, filtered, evaporated, and purified by column chromatography to give the peracetylated intermediate **H3** as a light-yellow solid in 53% yield. ^{1}H NMR (500 MHz, $CDCl_3$) δ/ppm=8.55-8.06 (24H, m), 7.21-6.65 (12H, m), 6.18-6.05 (12H, m), 5.51-5.29 (24H, m), 4.90-4.69 (24H, m), 4.31- 4.11 (36H, m), 3.77-3.01 (12H, m), 2.55-2.38 (12H, br), 2.15-1.90 (108H, m), 1.65-1.18 (36H, m); ^{13}C NMR (125 MHz, $CDCl_3$+CDOD) δ 175.25, 174.11, 149.29, 128.35, 119.93, 90.50, 79.29, 73.02, 66.59, 59.17, 57.58, 53.76, 34.33, 25.39. HRMS: m/z calcd for : m/z [M +$HCOO^-$] $C_{246}H_{300}N_{48}O_{108}$: 5698.9440, found 5699.3560.

2. *Cell culture*[4]

Jurkat and K562 cells were cultured in RPMI 1640 medium supplemented with 10% (v/v) FBS, and 1% penicillin-streptomycin at 37 °C with 5% CO_2 and 95% humidity. To prepare cell suspensions, Jurkat or K562 cells in logarithmic growth phase were removed from the culture medium by centrifugation. The cell density was diluted to 1.4×10^6 cells/mL after three washes with PBS. The modified QCM chips were placed in a 24-well plate, and 0.5 mL of the above cell suspension (containing 7×10^5 cells) was added and incubated in an incubator for 1 h to fix them on the chip surface. After incubation, the unfixed suspension cells were removed by washing with PBS, and the cells fixed on the surface of the chip were stained to detect the cell coverage. Finally, the chips were mounted on the cell sensing chip holder and set aside for use.

3. *Preparation and characterizations of chip*

3.1 Preparation of PDA-based supramolecular carbohydrate chip

a) Chip Cleaning: Gold sensor chips (Attana AB) were sequentially cleaned by (i) soaking in 1% NaClO solution (3 × 2-3 min), (ii) rinsing with copious amounts of ultrapure water, and (iii) treating with piranha solution (3:1 v/v concentrated H_2SO_4:30% H_2O_2) for 20 min. Caution: Piranha solution is highly corrosive and must be handled with extreme care. Chips were then rinsed with water until neutral pH, washed with ethanol, and dried under a gentle N_2 stream.

b) Polydopamine (PDA) Coating: A 2 mg/mL dopamine hydrochloride solution was prepared in 10 mM Tris-HCl buffer (pH 8.5). A volume of 50 µL of this solution was drop-casted onto each cleaned gold chip and allowed to self-polymerize at room temperature in a humid environment for 1.5 hours. The successful coating was indicated by a visible color change from bright gold to dark yellow/brown. Chips were rinsed with water and dried.

c) Immobilization of Ferrocene Guest: The amine-terminated ferrocene guest molecule (d) was dissolved in PBS (pH 7.4) to a concentration of 10 mM. A 50 µL aliquot was applied to the PDA-coated chip and incubated at room temperature for 2 hours, enabling covalent conjugation via Michael addition/Schiff base reaction. The chip was then thoroughly rinsed with water and dried.

d) Oxidation and Host Assembly: The ferrocene-modified chip was treated with 15% H_2O_2 for 4 minutes to oxidize ferrocene to ferrocenium, rinsed with water, and dried. Subsequently, a 50 µL aliquot of the N-GlcNAc-pillar[6]arene host (10 mM in PBS) was applied and incubated for 2 hours at room temperature to allow host-guest complex formation. The chip was rinsed with PBS and stored at 4°C until use.

3.2 Characterizations

a) Atomic Force Microscopy (AFM): Measurements were performed in tapping mode over a 5×5 µm² area at three random locations per chip type (n=3). Data analysis confirmed the stepwise build-up of the film (Table S1). The root-mean-square roughness (Rq) increased from 1.32 nm (bare gold) to 20.4 nm (PDA chip) and finally to 49.9 nm (carbohydrate chip), while the maximum height increased from 4.9 nm to 86.5 nm and 194.8 nm, respectively.

Table S1. AFM measurement of chip 3D surface morphology : roughness and thickness comparison (n=3)

Parameter（nm）	Gold chip	PDA chip	Carbohydrate chip
Ra	1.04±0.15	15.8±2.1	39.5±4.8
Rq	1.32±0.19	20.4±2.7	49.9±6.1
Max height	4.9±0.7	86.5±10.5	194.8±21.2
Min height	0.3±0.1	9.5±1.8	10.7±2.5

b) Scanning Electron Microscopy with Energy-Dispersive X-ray Spectroscopy (SEM-EDS): Elemental analysis was performed at 15 kV accelerating voltage. The spectra for the PDA chip showed prominent Au (substrate), Si (quartz), C, O, and N signals. The final carbohydrate chip spectrum showed a clear increase in the Fe signal (confirming ferrocene attachment) and a significant enhancement of the C and N signals (confirming the successful immobilization of the pillar[6]arene-based glycocluster), as referenced in the main text (Fig. 1d-e).

3.3 Preparation of living suspension cell chips

a) Cell Capture: Jurkat or K562 cells in logarithmic growth phase were washed with PBS and resuspended at a density of 1.4×10^6 cells/mL. A 0.5 mL aliquot of this suspension (containing 7×10^5 cells) was added to cover the supramolecular carbohydrate chip placed in a 24-well plate. The plate was incubated at 37°C with 5% CO_2 for 1.5 hours. Non-adherent cells were removed by gentle washing with PBS. Cell immobilization and viability were verified by staining with Hoechst 33258 (5 μg/mL, 20 min) and observation under a fluorescence microscope (Olympus BX53). Images showing successful capture and good cell morphology are presented in the Fig. S9.

b) Chip Regeneration and Reuse: After QCM experiments, the cell chip was incubated with 0.5 mL of 20 mM mercaptoethanol in PBS (pH 7.4) for 30 minutes at room temperature. This reduces the ferrocenium ions, leading to dissociation of the host-guest complex and cell release. The chip was washed with PBS, and cell removal was confirmed by fluorescence microscopy. The chip could then be re-oxidized with H_2O_2, re-assembled with the pillar[6]arene host, and used to capture a fresh batch of cells, demonstrating its reusability. (Fig. S10)

3.4 QCM detection and data collection

The cell chip was prepared and inserted into the Attana Cell biosensor. A pH 7.4 PBS solution was used as the running buffer, and the chip was stabilized at a flow rate of 20 μL/min with a frequency change of less than 5 Hz in 600 seconds. To seal the uncovered glycosyl groups and PDA on the chip surface, a 100 μg/mL bovine serum albumin (BSA) solution was injected 3-5 times until the frequency no longer changed.

For kinetic experiments, different concentrations of lectin (100, 50, 25, and 12.5 μg/mL) prepared in PBS were introduced onto the chip surface for detection. Subsequently, a high concentration (300 mM) of the corresponding monosaccharide solution was injected to elute the lectin for 15 seconds, thus regenerating the cell chip. Other lectins were then injected to detect the level of different glycol units on the surface of Jurkat and other cells. Each lectin was tested at least three times to reduce errors. The resonance frequency changes of the suspension cell chip were measured and recorded in real-time by Attester software (Attana AB). The data of competition experiments were fitted with GraphPad Prism 8, and the experimental data of kinetics were fitted with Clamp XP.

3.5 Kinetic and Competition Data Analysis

a) Kinetic Analysis Procedure: The kinetic parameters reported in Table 1 of the main text were derived through a global fitting analysis. The procedure is detailed as follows:

1) Model Selection: All sensorgrams were fitted globally to a 1:1 Langmuir binding model incorporating a mass transport parameter. This model is the standard for analyzing biomolecular interactions on biosensor surfaces where diffusion may influence the observed binding rate. It is described by the set of differential equations accounting for the transport of analyte (lectin) to the surface and the reversible binding reaction: $A + B \rightleftharpoons AB$. 2) Global Fitting Strategy: For each lectin-cell pair (e.g., WGA & Jurkat), the sensorgrams from all four analyte concentrations (12.5, 25, 50, 100 μg/mL) were fitted simultaneously. This "global fit" constrains the kinetic rate constants (k_{on}, k_{off}) to be identical across all concentrations, while allowing the response magnitude (Rmax) to scale with concentration. This approach yields more robust and accurate parameters than fitting each concentration curve individually. 3) Fitting Workflow: The analysis was performed using the Attana Evaluation Software (ClampXP). The stable baseline preceding each injection was used as the starting point. The software iteratively adjusted the parameters k_{on}, k_{off}, the mass transport coefficient (k_t), and the maximum binding capacity (R_{max}) to minimize the sum of squared

differences (χ^2) between the experimental data and the theoretical curves for all concentrations at once. 4) Fit Validation: The appropriateness of the 1:1 binding model for our data was validated by two key criteria: 5) Visual Inspection: A consistent and close overlay of the fitted curves onto the experimental sensorgrams across the entire concentration range (as exemplified in main text Figure 3b). 6) Residuals Analysis: For each global fit, the residuals (the difference between each experimental data point and the fitted curve) were examined. A valid fit is characterized by residuals that are randomly distributed around zero, showing no systematic trends or patterns. This random distribution confirmed that the model adequately described the data without significant bias.

b) Competition Assay Data Processing: For the competitive inhibition experiment (main text Figure 3a), the raw response for each injection of the Con A/D-mannose mixture was first corrected by digitally subtracting the signal from a prior injection containing the corresponding concentration of D-mannose alone. This corrected response reflects only the specific binding of Con A in the presence of the competitor. The corrected response at each inhibitor concentration was then normalized as a percentage of the maximum binding observed in the absence of D-mannose (0 mM). The normalized dose-response data were fitted to a standard four-parameter logistic (inhibition) model (Equation S1) using GraphPad Prism 8 software to determine the half-maximal inhibitory concentration (IC_{50}).

3.6 Recycling chips

The cell chip was placed in a 24-well plate, and 0.5 mL of a 20 mM mercaptoethanol solution prepared in PBS was added. It was then incubated at room temperature for 30 min to allow the reduction of the oxidized ferrocene group. After this, the chip was disassembled with pillar[6]arene to remove the cells and achieve recycling of the chip. Subsequently, the mercaptoethanol solution was removed, and the chip surface was washed with PBS 2-3 times. The cells on the chip surface were stained with Hoechst 33258 and observed by fluorescence microscopy to ensure that the cells on the chip surface had been completely removed.

4. *Fitting equation*

The curve fitting was performed by Graphpad Prism 5 software using the following equation:

$$\Delta f = \Delta f\mathrm{mean} + (\Delta f_{\max} - \Delta f_{\min}) / 1 + 10^{(\log C - \log IC_{50})}$$
Equation S1

Where: Δf mean response frequency Hz; $\Delta f_{\max}$ intended to be the maximum Hz of the response frequency; Δf mean response frequency nadir Hz; representative inhibitor concentration M; IC_{50} mean half inhibitory concentration M.

5. *Figure S1-S11*

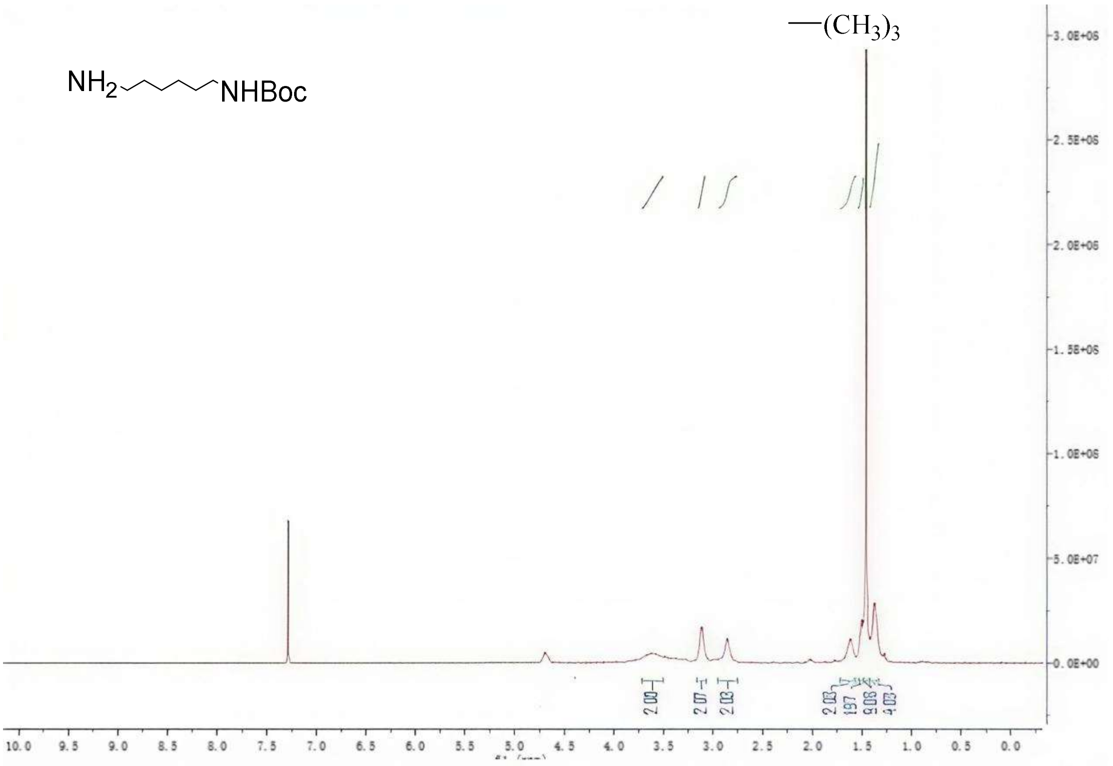

Figure S1. The ^{1}H-NMR Spectrum (500 MHz, $CDCl_3$) of Compound **G1**.

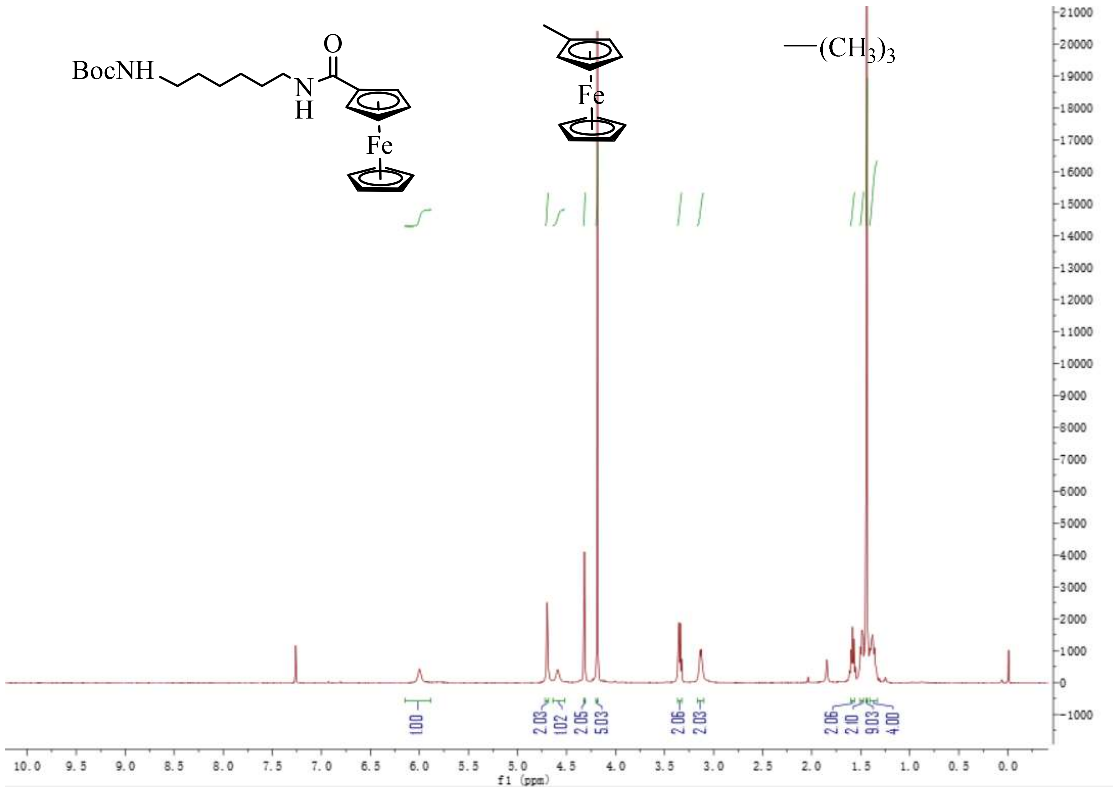

Figure S2. The ^{1}H-NMR Spectrum (500 MHz, $CDCl_3$) of Compound **G2**.

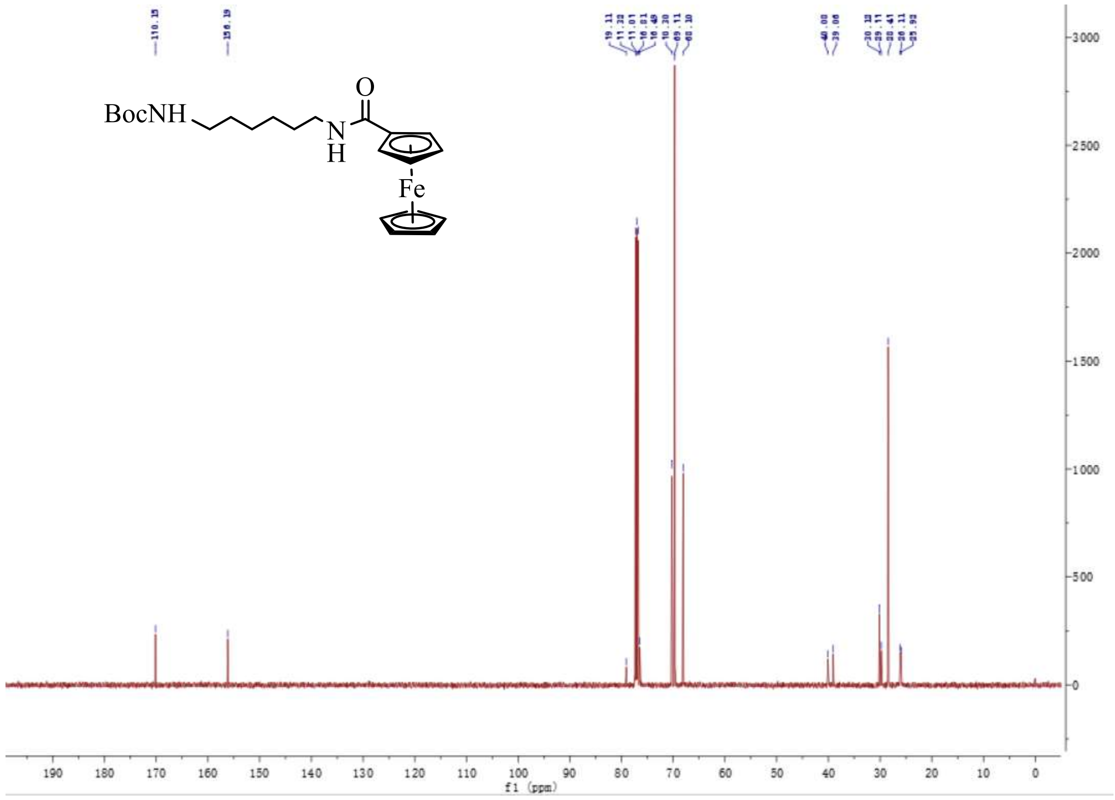

Figure S3. The ^{13}C-NMR Spectrum (125 MHz, $CDCl_3$) of Compound **G2**.

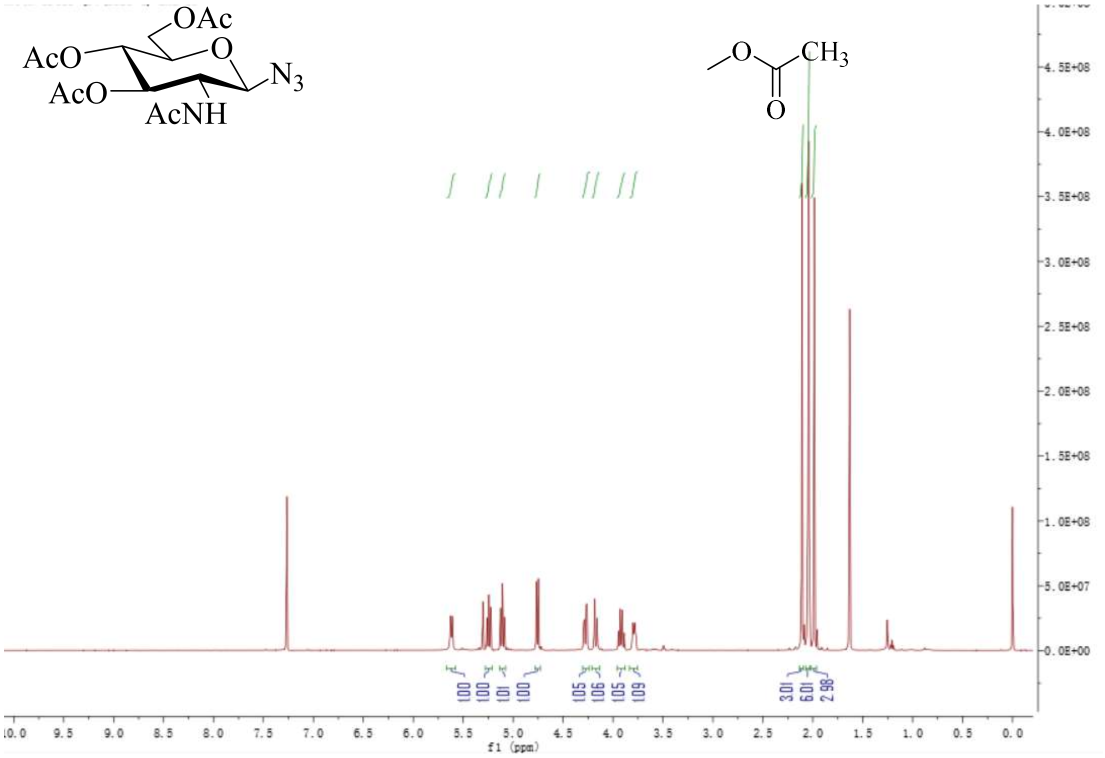

Figure S4. The ^{1}H-NMR Spectrum (500 MHz, $CDCl_3$) of Compound **H1**.

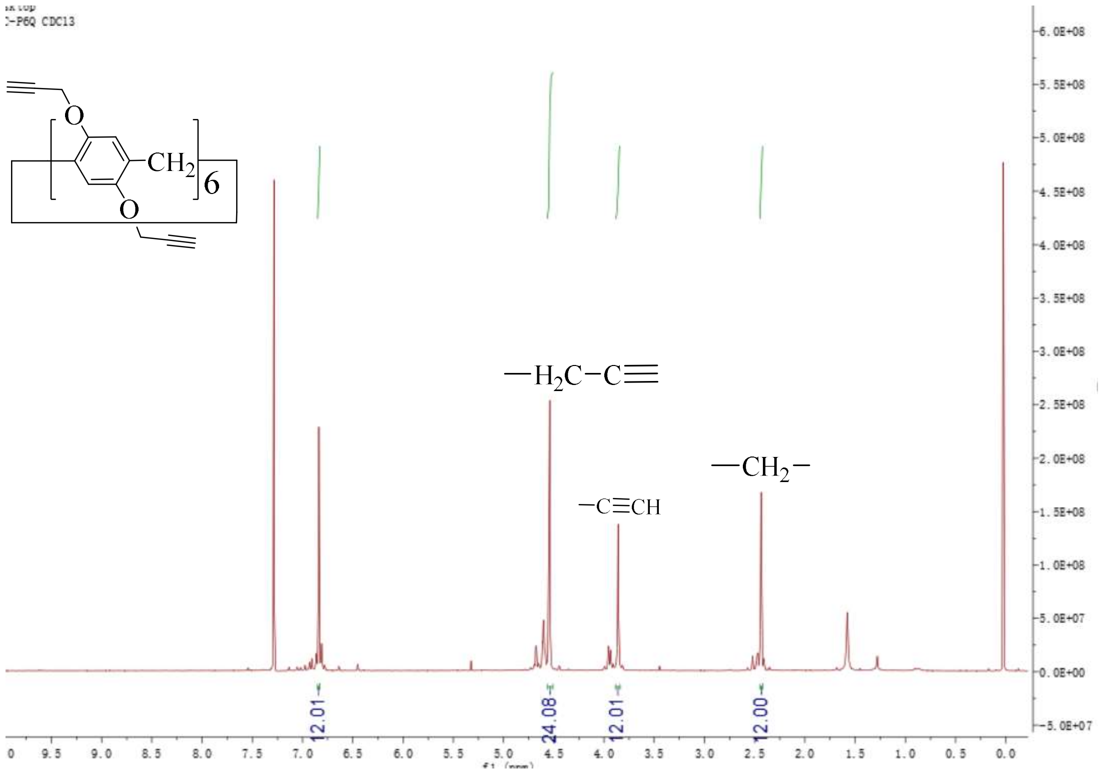

Figure S5. The ^{1}H-NMR Spectrum (500 MHz, $CDCl_3$) of Compound **H2**

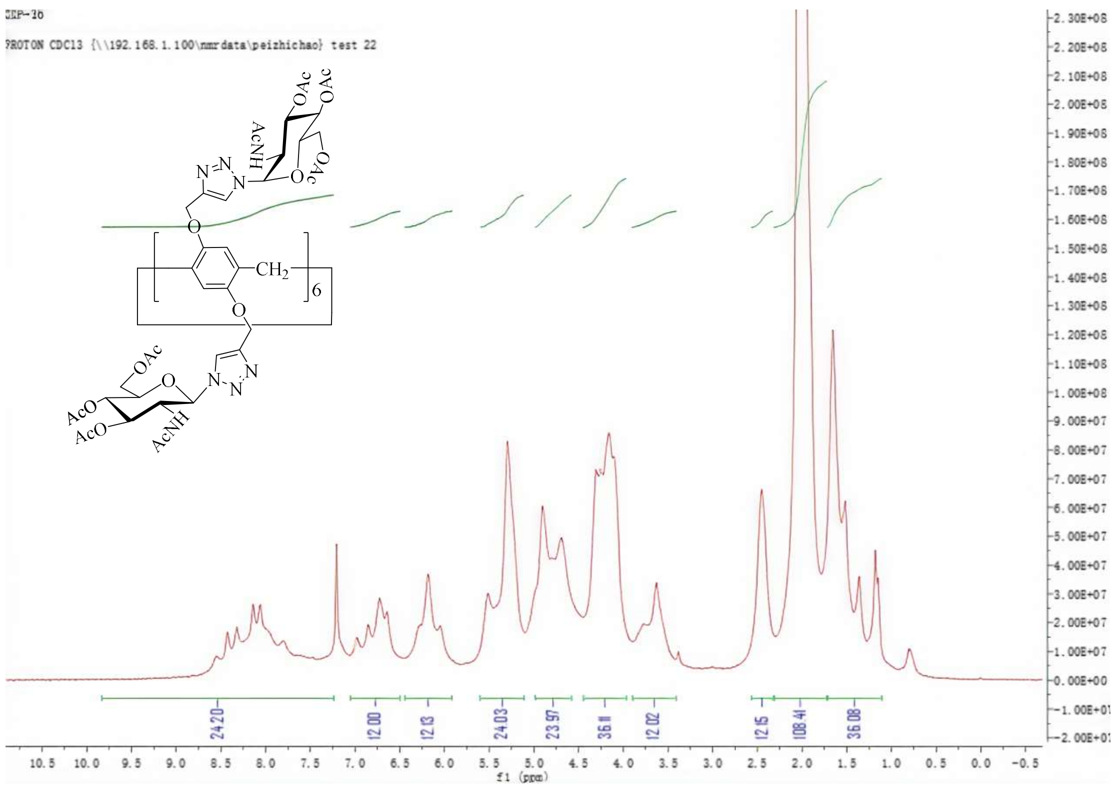

Figure S6. The ^{1}H-NMR Spectrum (500 MHz, $CDCl_3$) of Compound **H3**.

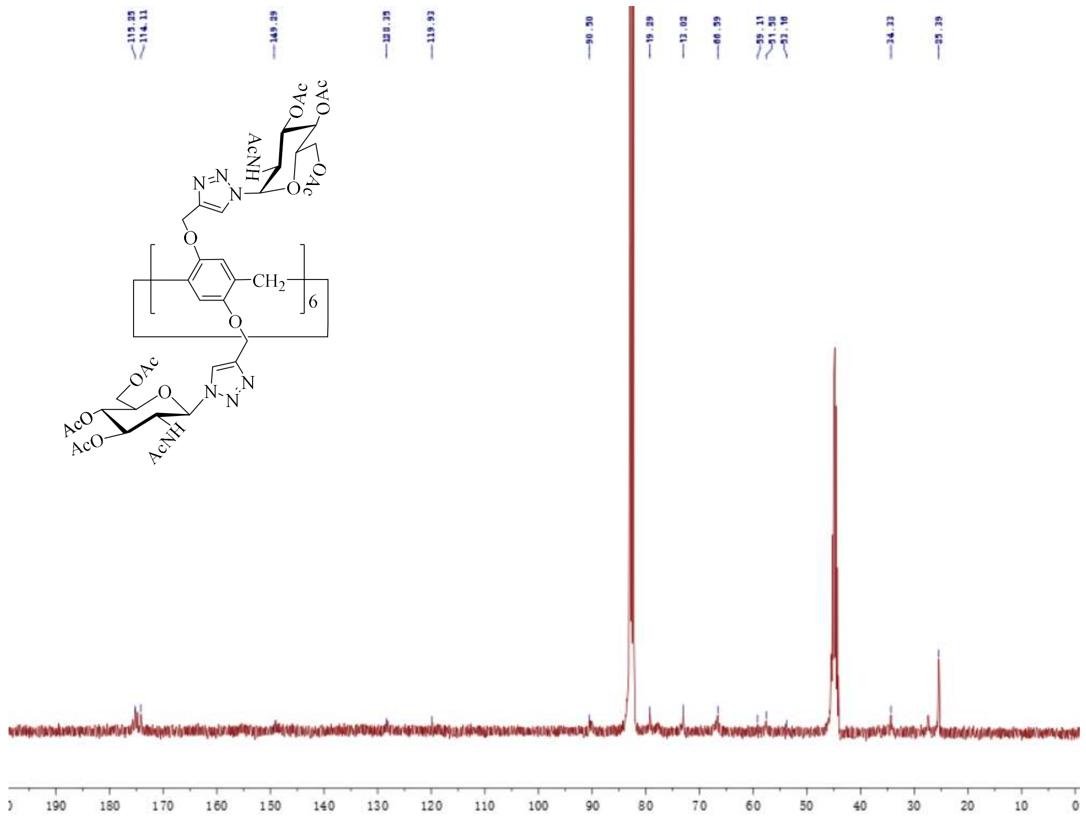

Figure S7. The ^{13}C-NMR Spectrum (125 MHz, $CDCl_3$) of Compound **H3**.

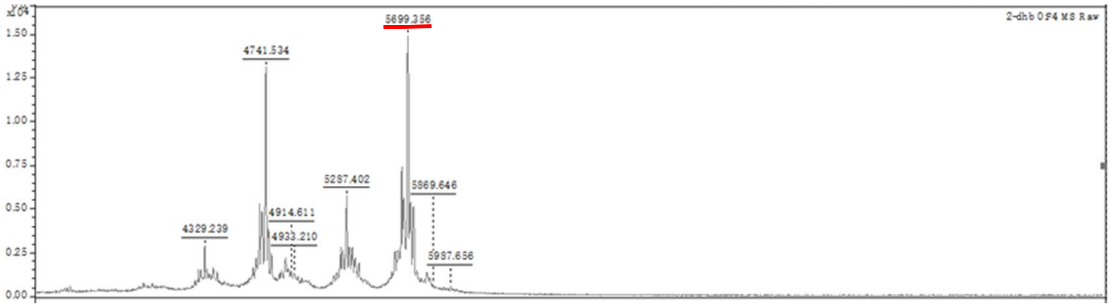

Figure S8. The HRMS Spectrum of Compound **H3**.

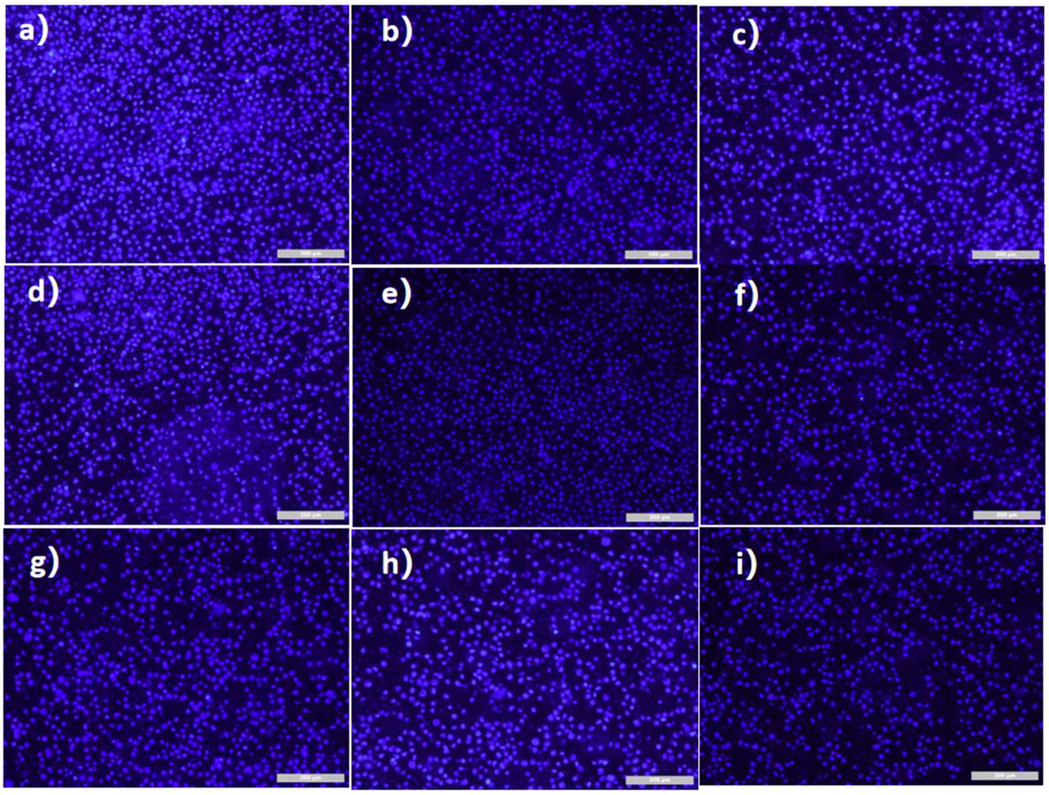

Figure S9 Fluorescence micrographs of Jurkat cells captured on the supramolecular glycochip before and after regeneration. (a-c) Three representative microscopic fields after the initial cell capture. (d-f) Three representative microscopic fields after the first regeneration and recapture cycle. (g-i) Three representative microscopic fields after the second regeneration and recapture cycle. Scale bar: 200 μm.

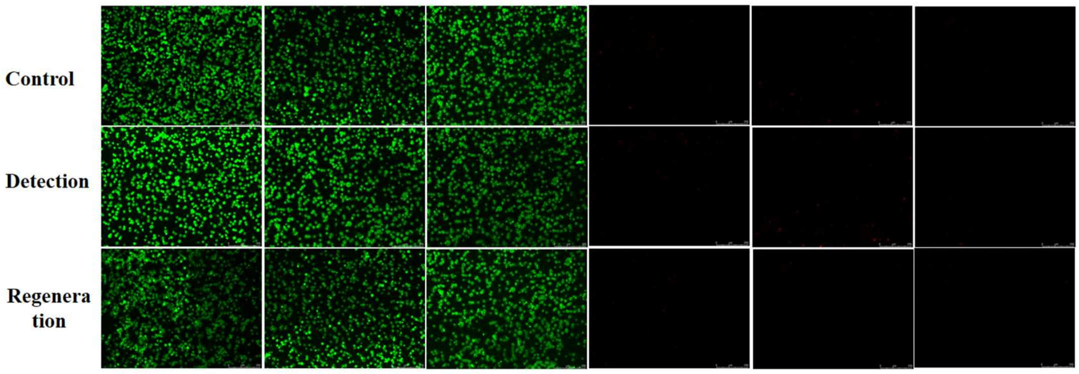

Figure S10 Live/dead fluorescence staining of Jurkat cells on the supramolecular glycochip across key processing stages. Images in each row correspond to a specific experimental group: Control (post-capture), Detection (post-detection), and Regeneration (post-regeneration & re-capture). For each group, the first three panels show live cell staining (green), and the subsequent three panels show dead cell staining (red). Scale bar: 200 μm.

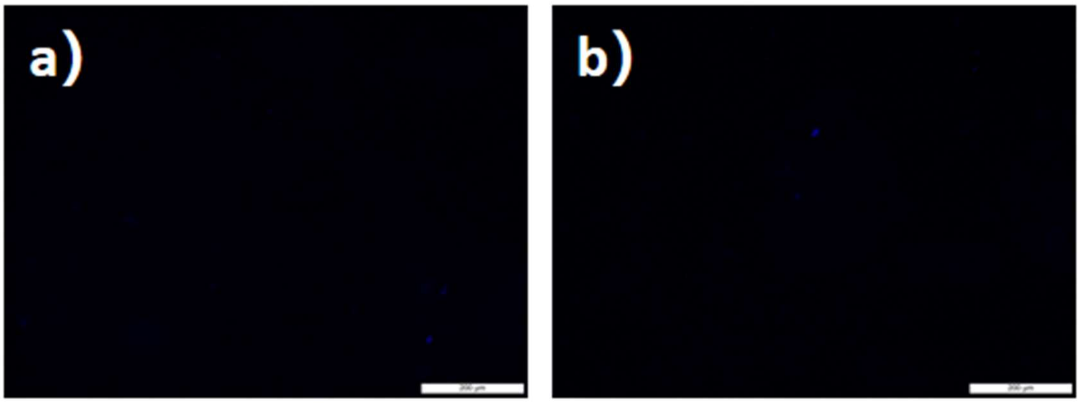

Figure S11 Fluorescence images of cells on (a) PDA-modified chip and (b) PDA–ferrocene-modified chip (without GlcNAc-P[6]). Scale bars: 200 μm.

Reference

[1] Brinkø, A.; Risinger, C.; Lambert, A.; et al. Combining Click Reactions for the One-Pot Synthesis of Modular Biomolecule Mimetics. *Org. Lett.* **2019**, 21, 7544–7548.

[2] Lu, Y.; Hou, C.; Ren, J.; Yang, K.; Chang, Y.; Pei, Y.; Dong, H.; Pei, Z. A multifunctional supramolecular vesicle based on complex of cystamine dihydrochloride capped pillar[5]arene and galactose derivative for targeted drug delivery. Int. J. Nanomed. 2019, 14 , 3525–3532.

https://doi.org/10.2147/IJN.S191256.
[3] Lu, Y.; Song, S.; Hou, C.; et al. Facile fabrication of branched-chain carbohydrate chips for studying carbohydrate–protein interactions by QCM biosensor. *Chin. Chem. Lett*. **2018**, 29, 65–68.
[4] Li, X.; Pei, Y.; Zhang, R.; et al. A suspension-cell biosensor for real-time determination of binding kinetics of protein–carbohydrate interactions on cancer cell surfaces. *Chem. Commun.* **2013**, 49, 9908–9910.
[5] Li, X.; Song, Q.; Pei, Y.; et al. *Direct attachment of suspension cells to PDA surface and its application in suspension-cell QCM biosensor.* **2021**, 326, 128823
